# Ultralow-threshold laser using super-bound states in the continuum

**DOI:** 10.1038/s41467-021-24502-0

**Published:** 2021-07-05

**Authors:** Min-Soo Hwang, Hoo-Cheol Lee, Kyoung-Ho Kim, Kwang-Yong Jeong, Soon-Hong Kwon, Kirill Koshelev, Yuri Kivshar, Hong-Gyu Park

**Affiliations:** 1 Department of Physics, Korea University, Seoul, Republic of Korea; 2 Department of Physics, Chungbuk National University, Cheongju, Republic of Korea; 3 Department of Physics, Chung-Ang University, Seoul, Republic of Korea; 4 Nonlinear Physics Center, Research School of Physics, Australian National University, Canberra, Australia; 5 School of Physics and Engineering, ITMO University, St Petersburg, Russia; 6 KU-KIST Graduate School of Converging Science and Technology, Korea University, Seoul, Republic of Korea

**Keywords:** Lasers, LEDs and light sources, Nanophotonics and plasmonics

## Abstract

Wavelength-scale lasers provide promising applications through low power consumption requiring for optical cavities with increased quality factors. Cavity radiative losses can be suppressed strongly in the regime of optical bound states in the continuum; however, a finite size of the resonator limits the performance of bound states in the continuum as cavity modes for active nanophotonic devices. Here, we employ the concept of a supercavity mode created by merging symmetry-protected and accidental bound states in the continuum in the momentum space, and realize an efficient laser based on a finite-size cavity with a small footprint. We trace the evolution of lasing properties before and after the merging point by varying the lattice spacing, and we reveal this laser demonstrates the significantly reduced threshold, substantially increased quality factor, and shrunken far-field images. Our results provide a route for nanolasers with reduced out-of-plane losses in finite-size active nanodevices and improved lasing characteristics.

## Abstract (web summary)

Though laser action has been reported for optical bound states in the continuum (BIC) cavities with high quality factors, these BIC lasers lacked practical applicability. Here, the authors report an ultralow-threshold super-BIC laser featuring merged symmetry-protected and accidental BICs.

## Introduction

Ultra-compact lasers operating in the single-mode regime have been a long-standing goal for nanophotonics. Various mechanisms for strong light confinement were proposed and demonstrated in subwavelength and wavelength-scale optical cavities used to decrease their lasing threshold^1–4^. However, nanolasers such as defect-type photonic crystal lasers and plasmonic lasers possess limited output power and exhibit instability to the structural disorder^5,6^. These properties hinder the practical applications of nanolasers although they have small mode volumes and reduced laser thresholds. On contrary, a band-edge type laser with a periodic structure without defects has a relatively high threshold, but a high output power with the possibility of topological robustness^7^.

Optical bound states in the continuum (BICs) were shown to be a versatile tool for substantial suppression of out-of-plane radiative losses and dramatic enhancement of the quality factor (Q factor) in infinite periodic structures to provide low-threshold lasing and high-output powers^8–10^. The intrinsic topological nature of BICs splits them into a few groups, with two of the most conventional kinds represented by symmetry-protected BICs, existing at high-symmetry points of the momentum space, and accidental BICs, which can be realized for an arbitrary in-plane wavevector. Thus far, symmetry-protected BICs and accidental BICs were observed successfully in Si_3_N_4_ and Si photonic crystal slabs^11,12^. In addition, the lasing action was achieved for BIC cavities in a few recent experiments^13–19^. The BIC-enhanced feature of a reasonably low threshold was successfully demonstrated in a cavity with lattices of finite size^13^. However, most of the developed lasers were still based on cavities of substantially large scales with hundreds of periods and despite that, they did not demonstrate high Q factors. Very recently, a new kind of BIC mode was suggested, which we term here as a *super-BIC* (BIC in the supercavity regime with extremely high values of the Q factor), that originates from the merging of several BICs in the momentum space^20,21^. The performance of super-BICs for active devices, however, was never discussed or studied.

Extended periodic structures consisting of hundreds of unit cells are feasible mostly for passive photonic devices^11,12,20^. For active devices such as a laser, however, it is critical to use a finite structure with a small footprint due to the limited spot size in optical or electrical pumping. For photonic crystal slabs with just a few dozen periods, the Q factor of conventional BICs is significantly reduced^22–25^. Thus, in the most of available studies, the size of BIC cavities was assumed to be infinite for analysis, which caused the difference between the experimental and theoretical results. In addition, it remains unclear whether the topological properties of BIC can be maintained in a finite-size cavity.

Here we demonstrate efficient lasing in a finite periodic photonic structure for three different regimes using one design: the symmetry-protected BIC laser, accidental BIC laser, and the super-BIC laser which is a combination of the first two BIC lasers, achieved by tuning the lattice constant. We achieve lasing action from small-scale dielectric photonic crystal slabs operating in the super-BIC regime. Our analysis shows that in the vicinity of the super-BIC mode the finite-size cavity can possess a high radiative Q factor, in contrast to the other BIC modes. We also show that for finite-size resonators the optimal lattice constant providing the lowest radiation is shifted compared to the infinite-size structure. After the transition to the super-BIC regime, we measure the far-field laser image with strong angular confinement, the reduced threshold to ~1.47 kW/cm^2^, and the increased Q factor up to ~7300. The measured threshold peak power is approximately 50 to ten million times lower than that of earlier demonstrated BIC nanolasers^13–19^. Our study presents the direct observation of all the BIC lasers simultaneously and provides an efficient recipe to reduce the optical loss in active nanocavities with finite sizes.

## Results

### Infinite-size BIC cavity

**Fig. 1:**
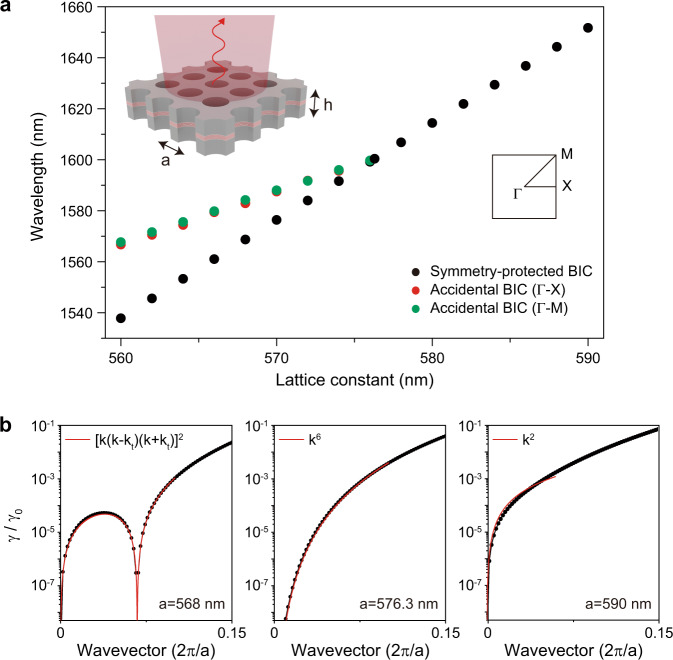
Merging of BICs in the infinite-size structure. **a** Calculated wavelengths of the symmetry-protected BIC (black dots) and the two accidental BICs (red and green dots) as a function of the lattice constant. The merging of the BICs occurs at *a* = 576.3 nm. The inset shows the schematic of the laser structure: the lattice constant is *a* and the slab thickness is *h*. **b** Calculated radiation loss (γ) normalized by the value near the light cone (γ_0_), γ/γ_0_, along the ΓX-direction wavevector. Three lattice constants are examined (from left to right): *a* = 568 nm (before merging), 576.3 nm (at merging), and 590 nm (isolated). The calculated curves (black dots) are fitted by [*k* (*k* – *k*_*t*_) (*k* + *k*_*t*_)]^2^, *k*^6^, and *k*^2^ (red curves), at *a* = 568, 576.3, and 590 nm, respectively.

The design of the laser cavity is based on an infinite-size InGaAsP photonic crystal slab structure with a thickness of 650 nm (inset in Fig. 1a) modulated with a square-lattice array of air holes. The calculated band structure shows that only the fundamental transverse-electric-like (TE-like) band is located within the emission wavelength range of the InGaAsP, ~1.5 μm, due to the thick slab of high-index material (Supplementary Fig. 1). The calculated map of Q factor in the *k*-space shows that the fundamental TE-like band demonstrates high Q factors at the origin and at specific points along highly symmetric Γ-X and Γ-M directions (Supplementary Fig. 2): in total, one symmetry-protected BIC (|**k**|= 0) and eight accidental BICs (*|***k**|~ *k*_*t*_ = 0.067*a*/2π) are formed^26^. In addition, the multipolar decomposition of electromagnetic fields inside the unit cell shows that both symmetry-protected and accidental BICs are dominated by the out-of-plane magnetic dipole, which agrees well with the fundamental nature of the TE-like mode (Supplementary Fig. 3). Each of accidental BICs is formed because of destructive interference between the dominating out-of-plane magnetic dipole and weak in-plane electric dipole, in-plane electric quadrupole, out-of-plane magnetic quadrupole. We also calculate the evolution of the polarization phases in the far-field for symmetry-protected and accidental BICs (Supplementary Fig. 4)^26^. The calculation shows the topological charge *q* = 1 for the symmetry-protected BIC and four accidental BICs located on the Γ-M band, and *q* = –1 for four accidental BICs located on the Γ-X band.

In addition, we calculate the wavelengths of the resonant modes shown in Fig. 1a. A symmetry-protected BIC and an accidental BIC mode appear in the range of the lattice constants *a* from 560 to 590 nm. The wavelengths of these modes become closer as the lattice constant increases at *a* < 576.3 nm, and only a single mode is observed after the two BIC modes merge at *a* = 576.3 nm.

Next, the normalized radiation loss of the fundamental TE-like band is calculated for different lattice constants along the Γ-X direction (black dots in Fig. 1b). At *a* = 568 nm, the zero-radiation loss is observed at *k* = 0 (symmetry-protected BIC) and at *k* = *k*_*t*_ (accidental BIC), showing each sharp dependence on the wavevector. The position of the accidental BIC approaches *k* = 0 as the lattice constant increases; the merging of the BICs occurs at *a* = 576.3 nm and modifies the dependence on the wavevector in the vicinity of the band edge. The *k*-vector dependence returns to the narrow one at *a* = 590 nm when the single symmetry-protected BIC is restored. The radiation loss follows the laws [*k* (*k* – *k*_*t*_) (*k* + *k*_*t*_)]^2^, *k*^6^, or *k*^2^ depending on the lattice constant (red curves in Fig. 1b), because of the interaction of the topological charges *q*_0_ at the symmetry-protected BIC and *q*_*t*_ at the accidental BIC^20^. This feature is also seen in the graph of the *k* vs. Q factor (Supplementary Fig. 1b).

### Finite-size BIC cavity

**Fig. 2:**
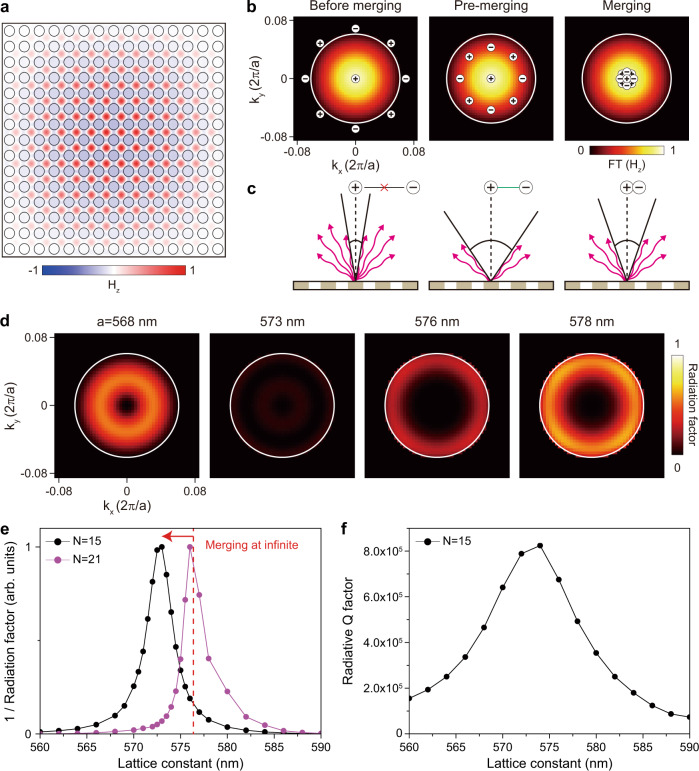
Merging of BICs in the finite-size structure. **a** Calculated H_z_ field distribution at *a* = 573 nm in the finite-size domain with *N* = 15. *N* is the number of air holes along the vertical (or horizontal) direction. **b** Topological charge distributions in FT(H_z_) at before-merging (left), pre-merging (middle), and merging (right). FT denotes the spatial Fourier transformation. The white circle of 7° indicates the first field minimum. **c** Schematic illustrations of the radiative loss in the three cases corresponding to **b**. **d** Calculated radiation factor, defined as |FT(H_z_)/Q | , for *a* = 568, 573, 576, and 578 nm. The largest dark area is obtained at pre-merging of *a* = 573 nm. **e** The values of the inverse radiation factor plotted as a function of the lattice constant for *N* = 15 (black) and *N* = 21 (purple). The vertical red dashed line indicates the merging point in the infinite-size domain. **f** Radiative Q factor for *N* = 15 as a function of the lattice constant, calculated by the FDTD simulation.

The fast sixth-order dependence of the radiation losses on the wavevector is crucial to keep a high Q factor for the BIC mode in a finite-size cavity^20^. Fig. 2a shows the mode magnetic field profile H_z_ at the Γ point of the fundamental TE-like band in a photonic crystal slab with 15 × 15 periods. Unlike the infinite-size cavity, the mode profile shows an envelope distribution with a convex shape accompanied by mode leakage into free space^25,27^. This finite-size effect results in the mode broadening Δ*k* (white circle) in the *k*-space field distribution, FT(H_z_) (Fig. 2b), where FT means the spatial Fourier transformation. The mode broadening leads to increased radiation loss due to mixing with the off-Γ point modes with a finite Q factor. To increase the Q factor, it is essential to reduce the radiation loss due to the broadening (Fig. 2c). The undesired radiation loss at off-Γ points can be effectively suppressed by moving the off-Γ BIC with the charge *q*_*t*_ inside the mode broadening range Δ*k* (pre-merging regime) or to the Γ point *k* = 0 (merging regime). Notably, in the merging process with a limited number of air holes, the most effective radiation suppression can be achieved by placing the charge *q*_*t*_ at an optimum *k*_*t*_ in the pre-merging configuration rather than at *k* = 0 (Fig. 2c), which is explained below.

To elucidate the radiation loss mechanism in the finite-size system, we calculate the 2D maps of the radiation factor in momentum space with varying lattice constants (Fig. 2d). The radiation factor is defined by the *k*-space mode distribution in a finite domain and the Q factor in an infinite domain, i.e., |FT(H_z_)(**k**)/Q(**k**)|, to account for both effects of the cavity size and the radiative loss. For example, the radiation factors are high before merging (*a* = 568 nm) and after merging (*a* = 578 nm) because of the substantial radiative loss at off-Γ points. At the exact merging condition of *a* = 576 nm, radiation loss still occurs at the boundary of the mode broadening. Interestingly, a much lower radiation factor is observed in the pre-merging regime at *a* = 573 nm because the radiative loss is strongly suppressed in the large *k*-space area covered by *q*_0_ and *q*_*t*_. For more detailed quantitative analysis, we plot the inverse radiation factor, [Σ | FT(H_z_)(**k**)/Q(**k**)|]^−1^, as a function of the lattice constant when the hole diameter and slab thickness are fixed to 400 and 650 nm, respectively (Fig. 2e). The broad merging configuration occurs due to the finite-size effect. The graph becomes narrower and the maximum occurs at a larger lattice constant when the cavity size increases from *N* = 15 to 21. Further discussion is performed to investigate the influences caused by other structural parameters on the radiation factor (Supplementary Fig. 5).

In addition, we calculate the radiative Q factor using a full-wave numerical simulation (Fig. 2f). The agreement between the radiative Q factor and the inverse radiation factor demonstrates the effectiveness of our analysis based on the radiation factor. Consequently, in the finite-size cavity, the radiative loss near the merging-BIC regime (from pre-merging to merging) is still low, whereas the loss in the other BIC regimes is relatively high. Also, the optimal point with the lowest radiation in the finite and infinite-size cavities could be different from each other (Supplementary Note 1).

### Measurements of BIC lasing

**Fig. 3:**
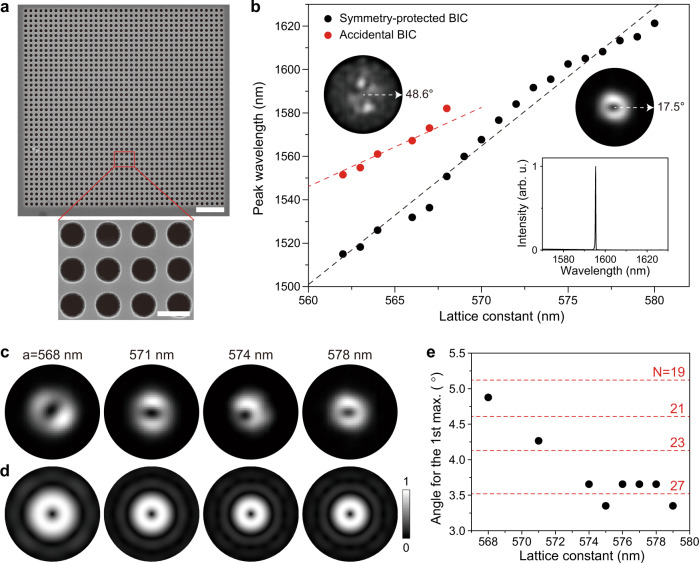
Characteristics of BIC lasers. **a** SEM images of the fabricated laser structure with *a* = 563 nm consisting of 40 × 40 unit cells. The scale bars are 3 μm (top) and 500 nm (bottom). **b** Measured lasing wavelengths of the symmetry-protected BIC lasers (black dots) and accidental BIC lasers (red dots) as a function of the lattice constant. The insets show the measured far-field images of the accidental BIC lasing mode (left; *a* = 568 nm) and the symmetry-protected BIC lasing mode (right; *a* = 578 nm). The bottom inset shows the above-threshold spectrum at *a* = 574 nm. Merging of the BICs occurs at *a* ~574 nm. **c** Measured far-field images of the symmetry-protected BIC lasers at *a* = 568 nm, 571 nm, 574 nm, and 578 nm (from left to right). The outer circle indicates 17.5°. **d** Simulated far-field images (|**E**|^2^) of the symmetry-protected BIC modes at *a* = 568 nm, 571 nm, 574 nm, and 578 nm (from left to right). The number of air holes (*N*) varies in the simulation (from left to right): *N* = 19, 23, 27, and 27. The outer circle indicates 17.5°. **e** Estimated angle for the 1st intensity maximum in the measured far-field images of the symmetry-protected BIC lasing modes (black dots). The red dashed lines indicate the calculated angles with varying *N*.

To experimentally verify the merging of the BICs, we fabricate square-lattice photonic crystal structures using a 650 nm-thick InGaAsP slab incorporating seven quantum wells (see “Methods” section). A set of samples with the lattice constant varying from 560 to 580 nm in 1 nm steps is fabricated with the hole radius fixed at ~200 nm (Supplementary Fig. 6). The scanning electron microscope (SEM) images of the fabricated sample are shown in Fig. 3a. The photoluminescence (PL) measurements are performed using a 980-nm pulsed pump laser with a spot size of ~5.4 μm at room temperature (see “Methods” section).

One or two types of lasing modes are observed in the photonic crystal structures, depending on the lattice constant. The measurement results of the lasing wavelength vs. the lattice constant in a sample are plotted in Fig. 3b. Single lasing peaks are observed at *a* > 568 nm (bottom inset in Fig. 3b) and two peaks at *a* ≤ 568 nm. The wavelengths of the two peaks increase with different slopes as the lattice constant increases at *a* ≤ 568 nm. To identify these lasing modes, we measure their far-field mode images (see “Methods” section). Two distinct far-field images are observed, one with a highly confined donut shape (right inset; Fig. 3b) and the other with a widespread shape (left inset; Fig. 3b). Consequently, the lasing peaks can be classified into the two groups of symmetry-protected BIC (black dots) and accidental BIC modes (red dots), based on the measured far-field images and a comparison with the simulation results including those presented in Fig. 1a. We note that the merging occurs at *a* ~574 nm by extrapolating the wavelength of the accidental BIC mode. Similar features are exhibited by the other samples with slightly different structural parameters (Supplementary Fig. 7).

To further investigate the optical properties of the symmetry-protected BIC laser, we compare the measured far-field images before merging (*a* = 568 and 571 nm) with those after merging (*a* = 574 and 578 nm) (Fig. 3c). The shapes of the lasing modes are identical, whereas the mode size decreases as the lattice constant increases and remains unchanged after merging. For more detailed quantitative analysis, we estimate the angle from the center to the first intensity maximum in eight measured far-field images (see “Methods” section). The change in the mode size is clearly shown in Fig. 3e (black dots). The angle decreases from ~4.9° to ~3.7° until *a* = 574 nm and remains almost constant at *a* ≥ 574 nm. In fact, the size of the far-field image depends on the mode size in the near field^28^. Thus, by comparing the sizes of the measured and simulated far-field images, one can estimate the effective number of air holes (*N*) in the photonic crystal structure for the excitation of the corresponding near-field image. Our numerical simulations show that the structures with *N* = 19, 23, 27, and 27 yields the measured far-field images in Fig. 3c (left to right in Fig. 3d). A more systematic comparison between the measurement and simulation is shown in Fig. 3e, where the red dashed lines indicate the simulation results. Therefore, in the evolution from before-merging to after-merging, we observe that the after-merging BIC mode is lasing with a larger effective *N*.

We term this BIC mode at *a* ≥ 574 nm the *super-BIC* mode, as it possesses optical characteristics such as a single lasing peak and a shrunken far-field image that are distinct from those of the conventional BICs at *a* < 574 nm. In particular, the super-BIC mode is confined more strongly by the effectively increased number of air holes. This unique feature is useful for improving the laser performance despite the effect of the finite size on the Q factor.

### Lasing properties

**Fig. 4:**
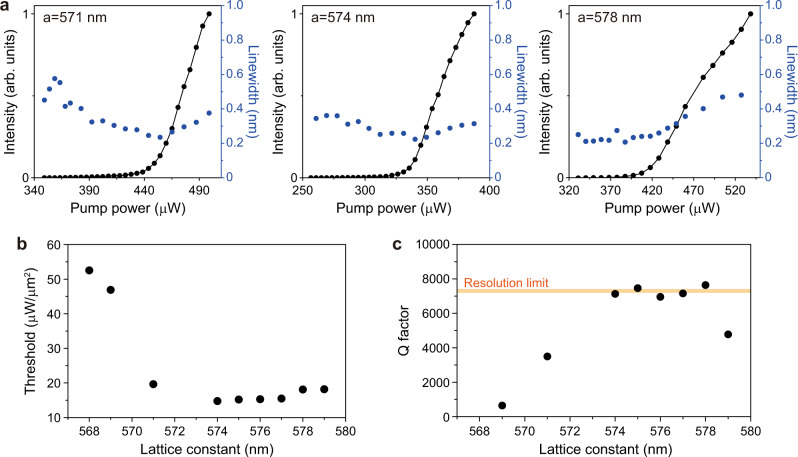
Threshold and Q factors of BIC lasers. **a** Measured L–L curves (black; left *y*-axis) and linewidth (blue; right *y*-axis) of the symmetry-protected BIC lasers at *a* = 571 nm, 574 nm, and 578 nm. The increase of the linewidth above the threshold is due to the thermal effect. **b** Measured threshold values divided by the pump area (~5.4 μm in size) as a function of the lattice constant. **c** Measured Q factors, λ/Δλ, as a function of the lattice constant. Δλ is the linewidth estimated at the transparent pumping condition (~340 μW). The linewidth data were taken from Supplementary Fig. 10. The orange line indicates the Q factor obtained using the resolution-limited linewidth of the spectrometer (~0.22 nm).

We examine the lasing properties of all demonstrated BIC lasers. First, the lasing spectra and light in–light out (L–L) curves are measured in the accidental BIC lasers (Supplementary Fig. 8); their threshold values are much larger than those of the symmetry-protected BIC lasers (Supplementary Fig. 9). Next, we measure the linewidth of the resonant peak and L–L curves in the symmetry-protected BIC lasers more systematically by varying the lattice constants (Supplementary Figs. 10 and 11). Fig. 4a shows representative measured data for three different lattice constants. Clear lasing behaviors are observed at the lasing thresholds of ~448, ~340, and ~413 μW at *a* = 571, 574, and 578 nm, respectively. Linewidth narrowing is also observed, although the below-threshold linewidth is small at *a* = 574 and 578 nm due to the high Q factor, which will be discussed in Fig. 4c. The threshold is much lower in the super-BIC regime at *a* = 574 nm. This feature is shown more clearly in the plot of the threshold power density vs. the lattice constant (Fig. 4b). The threshold decreases significantly as the lattice constant increases until *a* = 574 nm and becomes almost constant at *a* ≥ 574 nm. Thus, the measurement indicates that the lasing threshold is minimized by the transition to the super-BIC mode. We note that the threshold peak power of ~340 μW and threshold power density of ~14.7 μW/μm^2^ at *a* = 574 nm are the smallest among all the BIC lasers previously reported (Supplementary Table 1)^5,13–19,29^.

To understand the ultralow threshold of the super-BIC laser, we estimate the Q factor, λ/Δλ, where λ is the peak wavelength and Δλ is the linewidth of the peak at the transparent pumping condition at ~340 μW (see “Methods” section)^30^. The Q factors are plotted as a function of the lattice constant using all the linewidth data in Supplementary Fig. 10 (Fig. 4c). The Q factor increases with the lattice constant before merging but is almost constant after merging because Δλ is limited by the spectrometer resolution of ~0.22 nm. The experimental Q factor has a maximum value of ~7300 due to the spectrometer-limited linewidth, although the actual value is much higher. The maximum Q factor of ~7300 is exhibited from *a* = 574 to 578 nm, where the lasing threshold is also minimized. Therefore, the enhanced Q factor in the super-BIC regime is responsible for the significantly reduced threshold. We note that the effects of other factors such as mode volume and spontaneous emission factor on the threshold are not significant in the BIC laser with a relatively large mode volume^13^.

**Fig. 5:**
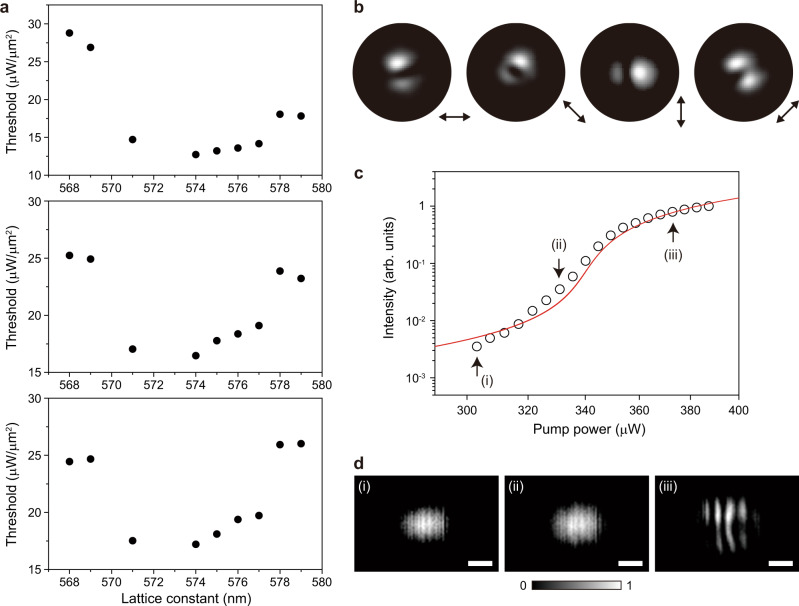
Lasing properties in the super-BIC regime. **a** Measured threshold values divided by the pump area as a function of the lattice constant, when the pump spot sizes are ~6.7 μm (top), ~8.0 μm (middle), and ~9.2 μm (bottom). **b** Measured polarization-resolved lasing images as a function of the polarizer axis. **c** Comparison of the measured L–L curve (open dots) with that obtained from the rate equations (red curve) in the log–log scale. The main parameters used in the rate equations are shown in “Methods” section. The estimated spontaneous emission factor is ~0.01. **d** Measured interference images in the near field, for (i) 303 μW, (ii) 331 μW, and (iii) 373 μW in the graph of **c**. (i), (ii), and (iii) correspond to the spontaneous emission, amplified spontaneous emission, and lasing regions, respectively. Scale bars, 10 μm. All data in **b**–**d** were measured in the laser structure with *a* = 574 nm.

Our measurements show that the super-BIC mode has a higher Q factor than the other BICs before merging, as indicated by Fig. 2. The effective increase in the number of air holes confining the super-BIC mode (Fig. 3e) further enhances the Q factor. The Q factor starts to decrease again at *a* = 579 nm (Fig. 4c), which indicates that the merging effect ends in this regime. This feature is more evident when the pump spot size increases (Supplementary Fig. 12). For larger pump spot sizes, the threshold values at *a* = 578 and 579 nm increase, whereas the super-BIC laser still shows a low threshold (Fig. 5a). In particular, the super-BIC occurs in a narrower range of lattice constants as the pump spot size increases, which is like the simulation result shown in Fig. 2e. Also, the super-BIC regime from *a* = 574 to 577 nm agrees well with the regime of calculated high radiative Q factors. Therefore, the merging point at *a* ~ 574 nm is further clarified through the experiment performed with varying pump spot sizes. At *a* = 578 and 579 nm, the lasing mode turns into the isolated BIC.

Furthermore, additional laser properties are investigated in the super-BIC regime (*a* = 574 nm). First, we measure the polarization-resolved lasing images by placing a linear polarizer in front of the IR camera (Fig. 5b). These images exhibit an intensity minimum along the direction of the polarizer, which agrees well with the previous report^31^. Second, we estimate the spontaneous emission factor, by comparing the measured L–L curve with that obtained from the conventional rate equations (Fig. 5c). The estimated spontaneous emission factor is ~0.01, which is smaller than the values of ultrasmall nanolasers^32^ due to the relatively large mode volume^33–35^. Third, the interference images are measured in the spontaneous emission, amplified spontaneous emission, and lasing regions in the super-BIC laser (Fig. 5d and “Methods” section). The interference pattern is clearly observed only in the lasing region, exhibiting the calculated coherence time of >38 ps (ref. ^36^). Fourth, we measure the decay times in the spontaneous emission and lasing regions of the super-BIC laser (Supplementary Fig. 13). The measured decay time of <138 ps in lasing is fast enough for the high-speed modulation^37,38^.

## Discussion

We have demonstrated a super-BIC laser based on a finite-size photonic cavity with a small footprint. We have observed a transition to the super-BIC laser from the symmetry-protected BIC and accidental BIC lasers by tuning the lattice constant. The theoretical analysis shows that the radiation loss in super-BIC follows the law *k*^6^ depending on the lattice constant and the super-BIC keeps a high Q factor even in the finite-size cavity. Thus, the high-performance optical characteristics, including an ultralow threshold, a single lasing peak, and a high Q factor, have been measured for the super-BIC laser. These features in the super-BIC laser are distinguishable from those in the symmetry-protected BIC laser. Notably, its threshold is extremely low and is limited by the transparency value of the gain material, as a result of the low radiative loss in the finite photonic structure. Furthermore, the semiconductor active material with high optical gain well supports the superior optical properties of super-BIC. Compared with other BIC lasers using similar gain materials (Supplementary Table 1), the threshold values we have measured are lower in the super-BIC regime and similar outside the super-BIC regime.

For the practical implementation of such an ultralow-threshold super-BIC laser, it is necessary to develop a flexible and stretchable laser structure^39^ to vary the lattice constant and find the merging point more easily. In addition, electrical pumping should be performed: an efficient current path needs to be formed to inject carriers to the whole area of the BIC cavity^7,40^. We believe that our findings will pave the way to significantly reduced optical losses in active nanophotonic structures with a finite footprint and the development of an ultralow-threshold light source for photonic integrated circuits, by controlling the topological charges in the reciprocal space and engineering the radiation condition.

## Methods

### Numerical simulations

The photonic band diagrams and optical properties of the resonant modes are calculated in a free-standing InGaAsP membrane using a three-dimensional finite-element method (FEM) solver in COMSOL Multiphysics. Floquet periodic boundaries and perfectly matched layers are imposed in the in-plane and vertical directions, respectively, for the infinite-size structures (Fig. 1 and Supplementary Figs. 1–4). The radiation loss (γ) is calculated by collecting the out-of-plane component of the Poynting vector away from the surface of the slab (Fig. 1b). Each radiation loss is normalized by the radiative loss at *k* = 0.275 (γ_0_), where the fundamental TE-like band is located near the light cone. The field decomposition into Cartesian multipoles (Supplementary Fig. 3) is done via the integration of the total field over the membrane volume within one unit cell. The topological charge is evaluated by integration of the polarization phase in the far-field domain over a closed loop in the *k*-space. For the simulation of the finite-size structures (Fig. 2), perfectly matched layers are introduced in all directions including the in-plane domain of *N* × *N* size. The far-field simulation is performed using a circular-shaped outer boundary to remove artificial interference patterns (Fig. 3d).

In addition, a home-made three-dimensional finite-difference time-domain (FDTD) method is used (Fig. 2f and Supplementary Fig. 5c) to calculate the radiative Q factor and cross-check the validity of the result in Fig. 2e, because FDTD can directly calculate Q factors in a finite-size cavity in the time domain by observing the time decay of resonant modes. The convolutional perfectly matched layer is used as an absorbing boundary condition in the FDTD. The size of the mesh grid is 10 nm, and more than 1000 periods of resonance oscillations are observed in the time domain to precisely calculate resonant wavelengths and Q factors. The Poynting vector is decomposed into in-plane and vertical components in the slab structure, and the radiative Q factor is then obtained using the vertical component.

In the FEM and FDTD simulations (except for Supplementary Fig. 5), the hole diameter and slab thickness are set to 400 and 650 nm, respectively. Different structural parameters are examined in Supplementary Fig. 5. The refractive index of the InGaAsP slab is set to 3.4.

### Device fabrication

The samples are fabricated using a 650 nm-thick InGaAsP/1 μm-thick InP/100 nm-thick InGaAs/InP substrate wafer. The InGaAsP layer includes seven 7 nm quantum wells in the middle, whose central emission wavelength is ~1.5 μm. The InP and InGaAs layers act as sacrificial and etch stop layers, respectively. To define a periodic square-lattice structure, electron-beam lithography is performed at 30 keV on a polymethyl methacrylate (PMMA) layer coated on the wafer. The hole diameter is fixed at ~400 nm and the lattice constant varies from 560 to 580 nm. Chemically assisted ion-beam etching is performed to drill air holes in the InGaAsP layer while using the PMMA layer as an etch mask. Finally, the sacrificial InP layer is selectively wet-etched using a diluted HCl: H_2_O (4:1) solution at room temperature, and the remaining PMMA layer on top of the slab is removed by O_2_ plasma.

### Optical measurements

A 980-nm pulsed laser diode (2.0% duty cycle, 1 MHz period) is used to optically pump the fabricated samples at room temperature. The light emitted from the cavities is collected by a ×100 objective lens with a numerical aperture of 0.85 (LCPLN100XIR, Olympus) and focused onto either a spectrometer with an IR array detector (SP 2300i and PyLoN, Princeton Instruments) or an InGaAs IR camera (C10633, Hamamatsu). The spot size of the pump laser is varied from ~5.4 to ~9.2 μm using additional bulk lenses. The resolution of the spectrometer is ~0.22 nm. In Fig. 3b, the wavelength is taken just above the threshold of each laser to minimize the thermal effect. In the insets of Fig. 3b and in Fig. 3c, the far-field images are measured using a 4-*f* system consisting of bulk lenses and a spatial filter. All the L–L curves and linewidth graphs are plotted as a function of the peak pump power.

In Fig. 5d, the Michelson interferometer setup is used to measure the interference images^36^. The beams from the two arms of the interferometer are overlapped in time and space. In Supplementary Fig. 13, time-resolved PL measurement is performed using a near-IR femtosecond fiber laser (FemtoFiber pro-NIR, Toptica Photonics) with a repetition rate of 80 MHz and a wavelength of 780 nm. The emitted photons are detected by a superconducting nanowire single-photon detector (Eos 210 CS, Single Quantum) and time tagger (Time Tagger Ultra, Swabian Instruments). In addition, we estimate the external differential quantum efficiency, ~1.2%, in the laser structure with *a* = 574 nm (Fig. 4a, middle). This value needs to be further improved through the optimization of the structure^40,41^, although it is higher than those in small-size nanolasers^42,43^.

### Data analysis

To accurately estimate the size of the donut-shaped far-field image (Fig. 3e), we measure the average distance between the center of the donut and the position where the intensity is maximized. This distance is converted to the angle by comparing the far-field image with the reference image corresponding to a numerical aperture of the objective lens we used.

The experimental Q factor in Fig. 4c is obtained by λ/Δλ, where λ is the peak wavelength and Δλ is the linewidth at the transparency pump power. The transparency pump power *L*_*tr*_ is estimated using the conventional rate equation^30^: *L*_*tr*_ = 1/*η* × ħ *ω*_*p*_ *V*_*a*_ *B N*_*tr*_^2^, where *η* is the absorbed ratio in the quantum wells, ħ is the Plank constant, *ω*_*p*_ is the frequency of the 980-nm pump laser, *V*_*a*_ is the active region volume, *B* is the radiative recombination coefficient, and *N*_*tr*_ is the transparency carrier density. The nonradiative recombination coefficients are ignored. Using *η* = 0.24, *V*_*a*_ = 1.12 × 10^−12^ cm^3^, *B* = 1.6 × 10^−10^ cm^3^/s, and *N*_*tr*_ = 1.5 × 10^18^ cm^−3^ for our InGaAsP quantum wells, we obtain *L*_*tr*_ ~ 340 μW. In addition, to estimate the spontaneous emission factor of the super-BIC laser (Fig. 5c), the logarithmic gain *g*(*N*) = *g*_0_ (*c*/*n*_*eq*_) ln(*N*/*N*_*tr*_) is assumed in the rate equations, where *N* is carrier density, *g*_0_ is the gain coefficient, *c* is the speed of light, and *n*_*eq*_ is the effective refractive index^30^. The following parameters are used for fitting: *g*_0_ = 3000 cm^−1^ (ref. ^44^) and *n*_*eq*_ = 2.3.

## Supplementary information

Supplementary Information

## Data Availability

The data that support the findings of this study are available from the corresponding authors upon request.

## Code availability

The codes used in this work are available from the corresponding authors upon request.
