# Supplementary material for: Ultralow-threshold laser using super-bound states in the continuum
Source: Nat Commun. 2021 Jul 5;12:4135. doi: 10.1038/s41467-021-24502-0 (PMC8257597; doi:10.1038/s41467-021-24502-0)
Supplement: Supplementary file 1 — Supplementary Information [file 41467_2021_24502_MOESM1_ESM.pdf]

Supplementary Information for

Ultralow-threshold laser using super-bound states in the continuum

Min-Soo Hwang[1†], Hoo-Cheol Lee[1†], Kyoung-Ho Kim[2†], Kwang-Yong Jeong[1],

Soon-Hong Kwon[3], Kirill Koshelev[4,5], Yuri Kivshar[4*], and Hong-Gyu Park[1,6*]

[1]Department of Physics, Korea University, Seoul 02841, Republic of Korea.

[2]Department of Physics, Chungbuk National University, Cheongju 28644, Republic of Korea.

[3]Department of Physics, Chung-Ang University, Seoul 06974, Republic of Korea.

[4]Nonlinear Physics Center, Research School of Physics, Australian National University, Canberra ACT 2601, Australia.

[5]School of Physics and Engineering, ITMO University, St Petersburg 197101, Russia.

[6]KU-KIST Graduate School of Converging Science and Technology, Korea University, Seoul 02841, Republic of Korea.

*Corresponding authors. Email: ysk@internode.on.net; hgpark@korea.ac.kr

†These authors contributed equally to this work.

This file includes:

Supplementary Figs. 1 to 13

Supplementary Table 1

Supplementary Note 1

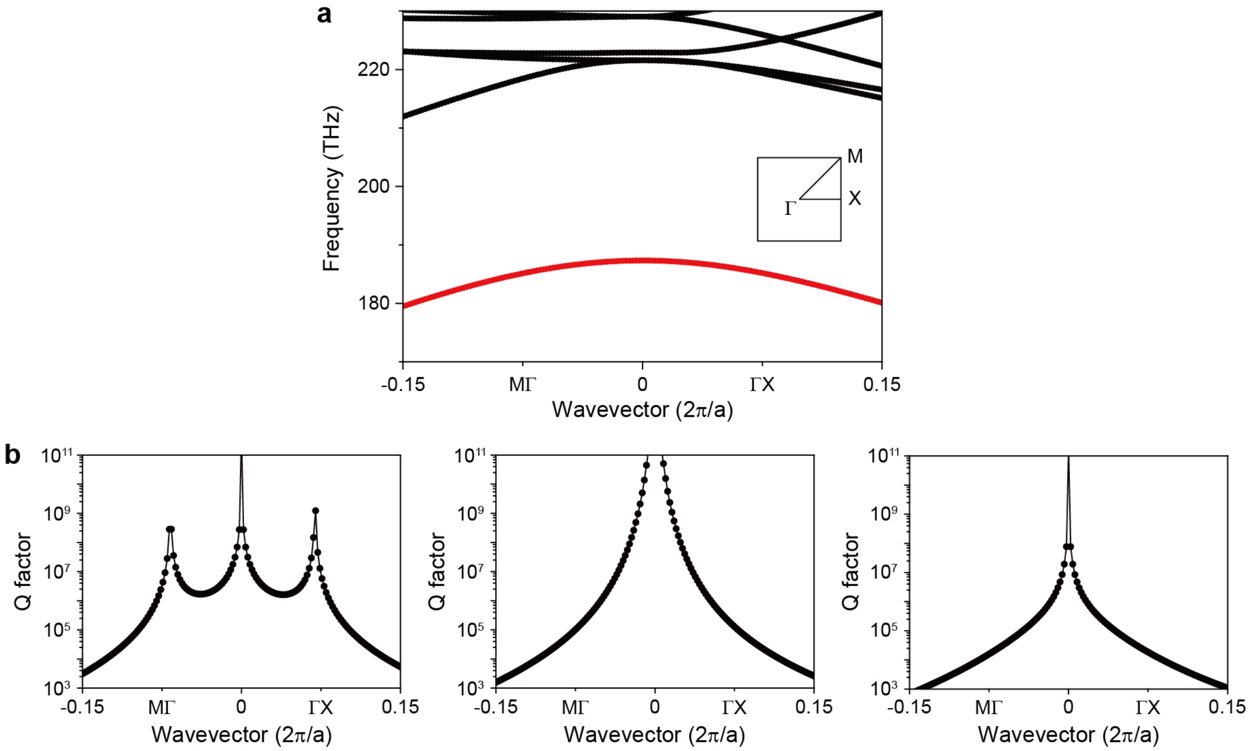

Supplementary Fig. 1. FEM simulation results for the infinite-size domain. a, Calculated TE-like band structure at a = 576.3 nm. Only the 1st band (red curve) is considered because its wavelength is well matched to the emission wavelength of the InGaAsP gain material. **b,** Calculated Q factors at a = 568 nm (left; before merging), 576.3 nm (middle; at merging), and 590 nm (right; isolated).

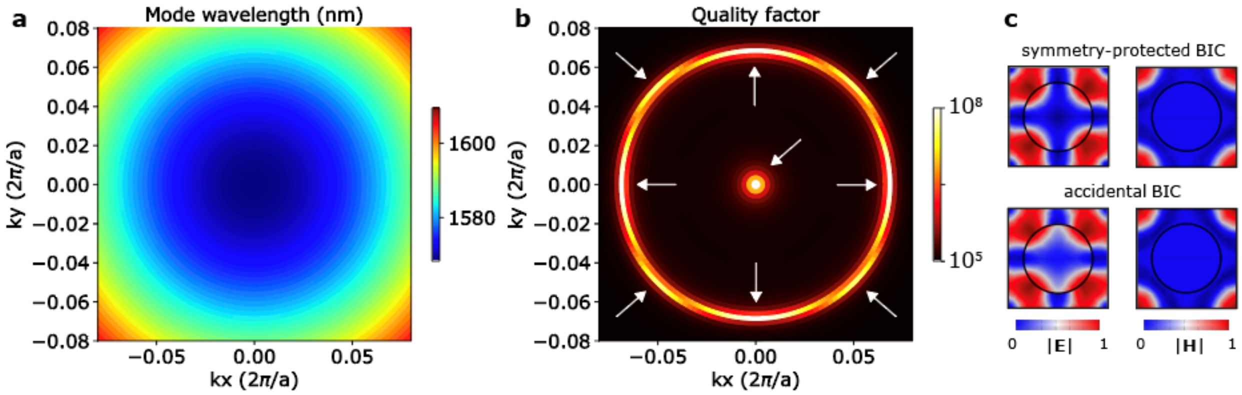

Supplementary Fig. 2. Fundamental TE-like mode dispersion for the infinite-size structure before merging. Calculated mode wavelength (**a**) and Q factor (**b**) for a = 568 nm in the momentum space. The mode wavelength increases away from the Γ point in **a**. The positions of symmetry-protected and accidental BICs in **b** are indicated by the gray arrows. Eight accidental BICs lie on a ring-shaped envelope at the distance $|\mathbf{k}| \sim 0.067a/2\pi$ from the origin. **c,** Calculated electric (left) and magnetic (right) field distributions of symmetry-protected and accidental BICs.

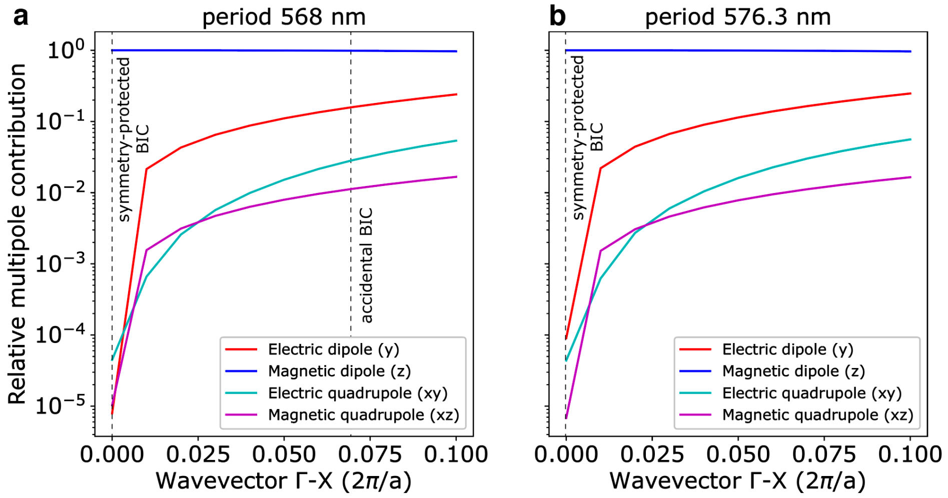

Supplementary Fig. 3. Multipolar analysis for the infinite-size structure before merging and at merging. Dominant multipolar contributions to electromagnetic fields in the unit cell for the fundamental TE-like band along the Γ-X direction in the k-space for a = 568 nm (**a**) and a = 576.3 nm (**b**). **a,** In the before-merging regime (a = 568 nm), the symmetry-protected BIC is dominated by the out-of-plane magnetic dipole component. For accidental BIC, the magnetic dipole interferes destructively with in-plane electric dipole, in-plane electric quadrupole, and out-of-plane magnetic quadrupole. **b,** In the merging regime (a = 576.3 nm), contribution of the electric dipole is increased compared to the before-merging regime. The photonic cavity structure possesses D_{4h} point symmetry at the Γ point. The multipolar analysis shows that the fundamental TE-like mode corresponds to A_{2g} irreducible representation, which transforms to B_2 irreducible representation along Γ-X and Γ-M.

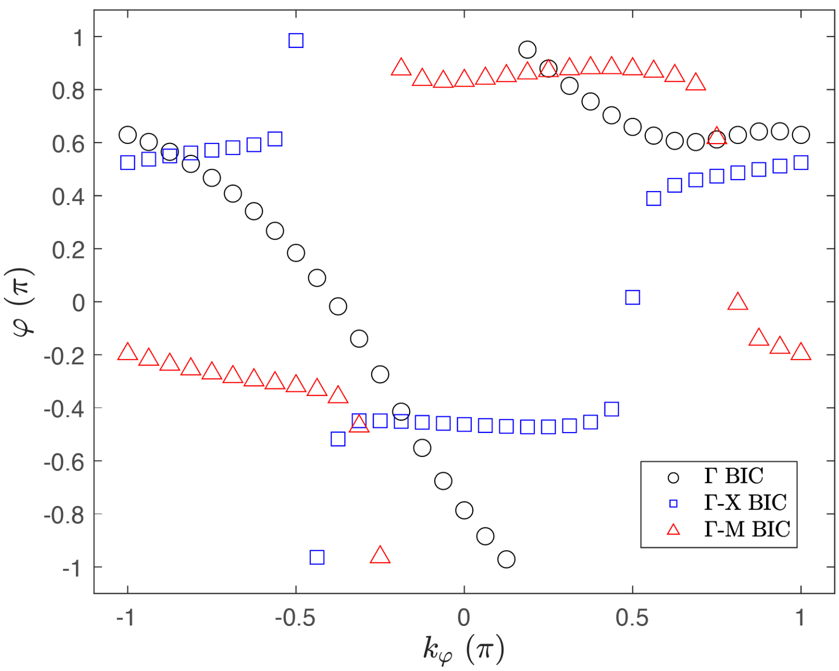

Supplementary Fig. 4. Polarization phase evolution in the far-field and topological charge calculation for the infinite-size structure for a = 568 nm. For each BIC, we choose a circle around it with $|\mathbf{k}| = 0.01a/2\pi$ and $k_\phi = [-\pi; \pi]$ as a closed path in k-space. Due to phase discontinuities, the integration along the path results in $q = 1$ for the symmetry-protected BIC and four accidental BICs located on the Γ-M band, and $q = -1$ for four accidental BICs located on the Γ-X band.

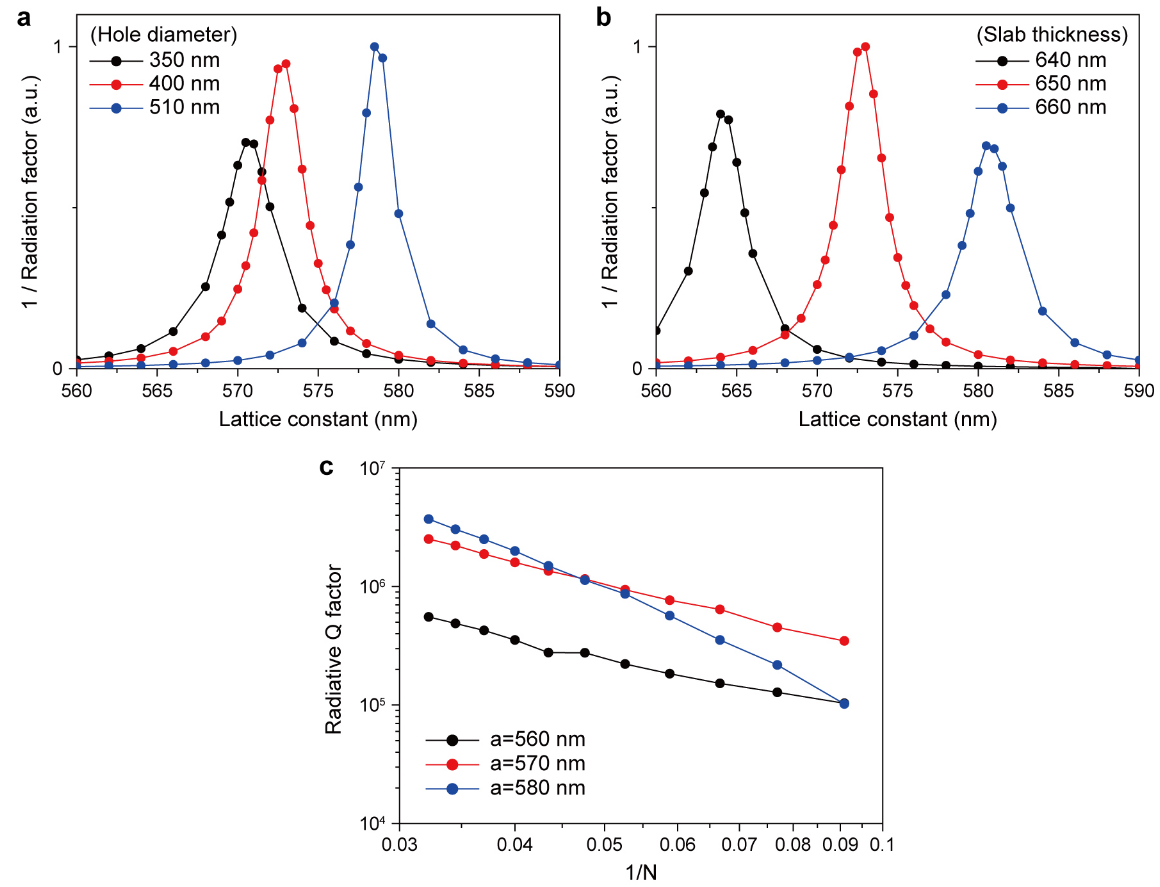

Supplementary Fig. 5. Inverse radiation factor and radiative Q factor for various structural parameters. a, Calculated inverse radiation factor as a function of the lattice constant, for different hole diameters of 350 (black), 400 (red), and 510 nm (blue). The slab thickness is fixed to 650 nm. The number of air holes along the vertical (or horizontal) direction, N, is 15. **b,** Calculated inverse radiation factor as a function of the lattice constant, for different slab thicknesses of 640 (black), 650 (red), and 660 nm (blue). The hole diameter is 400 nm and N is 15. The simulation results show that the inverse radiation factor increases with increasing hole diameter (a), while it is maximized in the optimized thickness of 650 nm (b). In addition, the thickness variation shows a rapid change of the merging point. We note that the position of accidental BIC varies in the momentum space with changing structural parameters, and thus, the merging point is changed accordingly. **c,** Calculated radiative Q factor as a function of $1/N$. The hole diameter and slab thickness are set to 400 and 650 nm, respectively. The lattice constant, a, is 560 (black), 570 (red), and 580 nm (blue). The radiative Q factor decreases as N decreases. However, the radiative Q factor remains high, $>3 \times 10^5$, at $N = 11$ near the super-BIC regime (a

= 570 nm), whereas it decreases rapidly with decreasing N outside the super-BIC regime (a = 560 and 580 nm). The calculated radiative Q factors at $N = 19$ and 11 (a = 570 nm) are ~3 times different. Since robust lasing was achieved at $N \geq 19$ in our experiment (Fig. 3e), we can expect lasing is also possible at $N \geq 11$ that is roughly the minimum number of unit cells for lasing. This estimation agrees with the previous result of BIC laser[13].

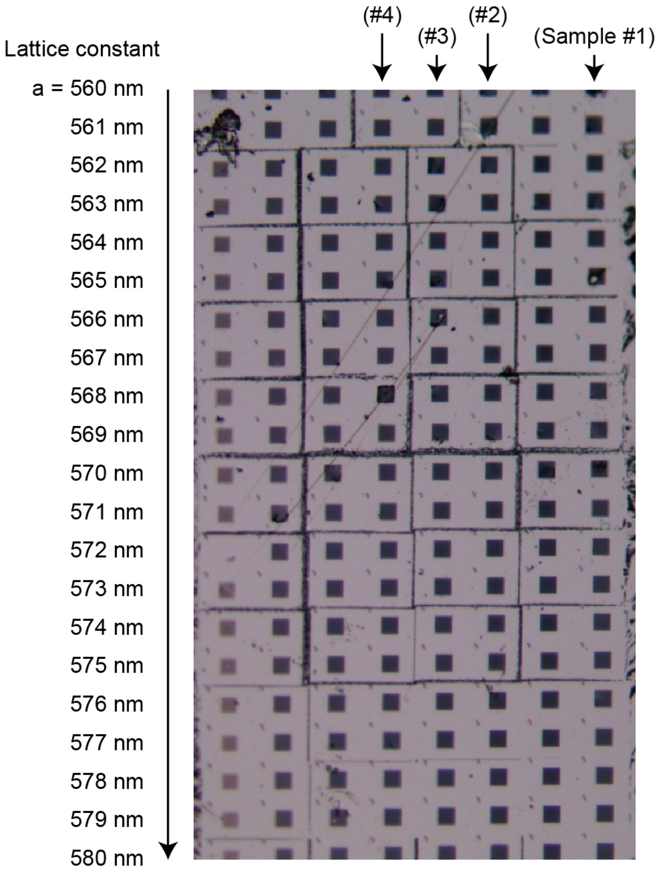

Supplementary Fig. 6. Fabricated samples with varying lattice constants from 560 to 580 nm. The lattice constant difference is 1 nm. We examine 4 samples: samples #1, #2, #3, and #4 (the same columns).

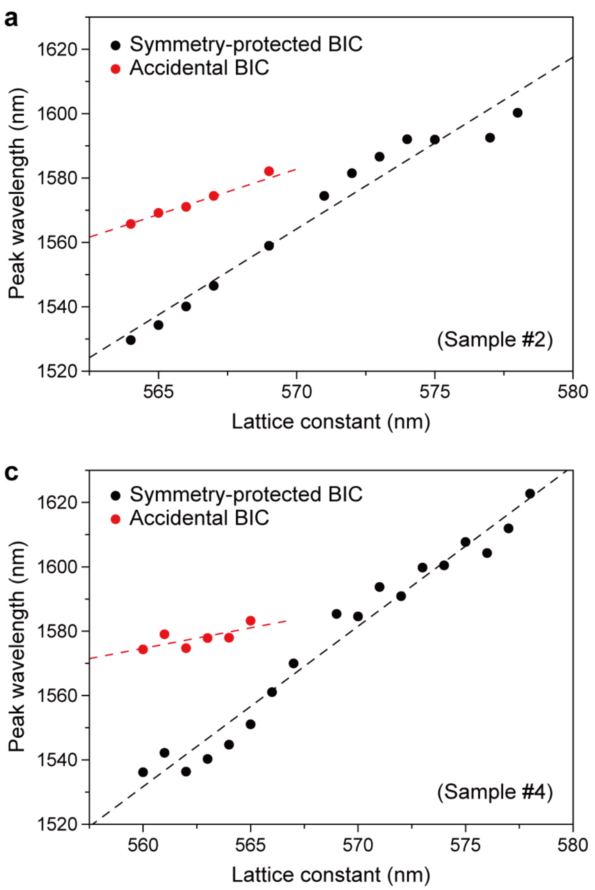

Supplementary Fig. 7. Measured peak wavelengths of the symmetry-protected BIC lasers (black dots) and accidental BIC lasers (red dots) as a function of the lattice constant for sample #2 (**a**), sample #3 (**b**), and sample #4 (**c**). Sample #1 is shown in Figs. 3 and 4.

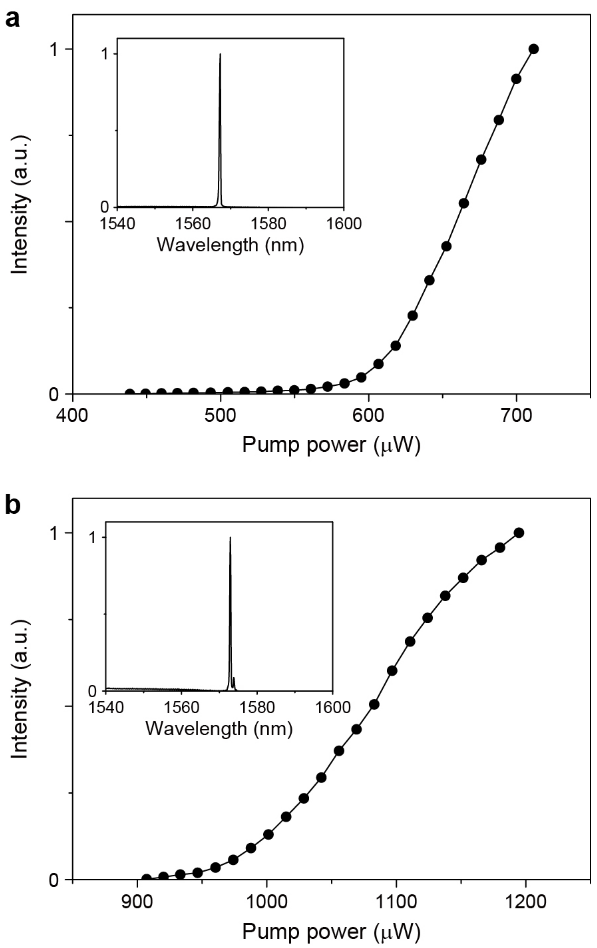

Supplementary Fig. 8. Measured L–L curves and above-threshold spectra (insets) of the accidental BIC lasers with a = 566 nm (**a**) and a = 567 nm (**b**) in Fig. 3b.

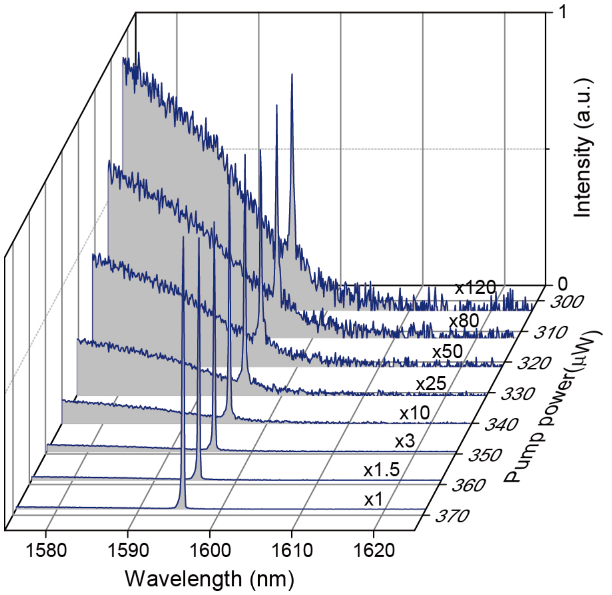

Supplementary Fig. 9. Measured PL spectra of the laser structure with a = 574 nm in Fig. 3 with varying pump power.

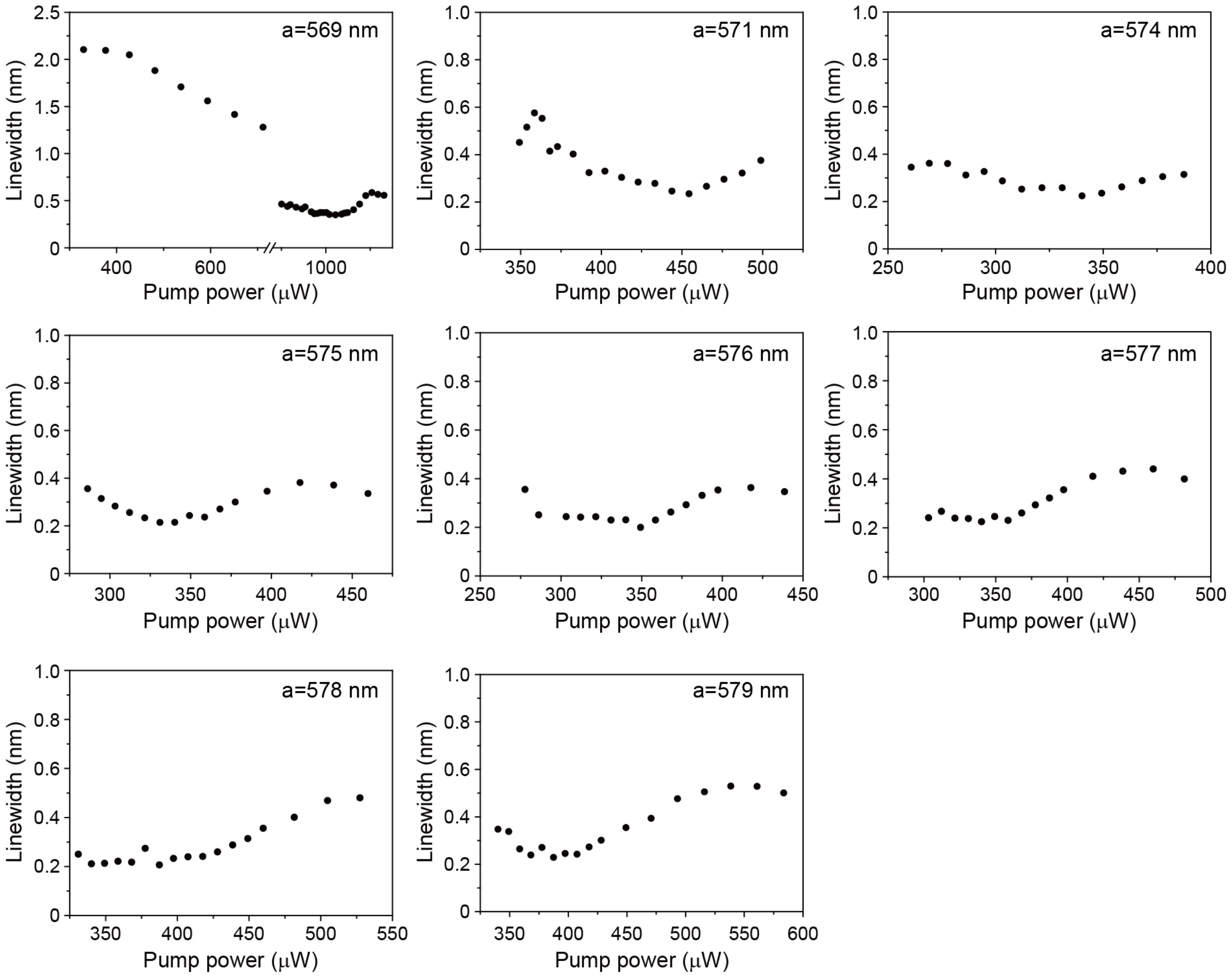

Supplementary Fig. 10. Measured linewidths as a function of the peak pump power in laser structures with different lattice constants. These data were used to estimate the Q factors in Fig. 4c.

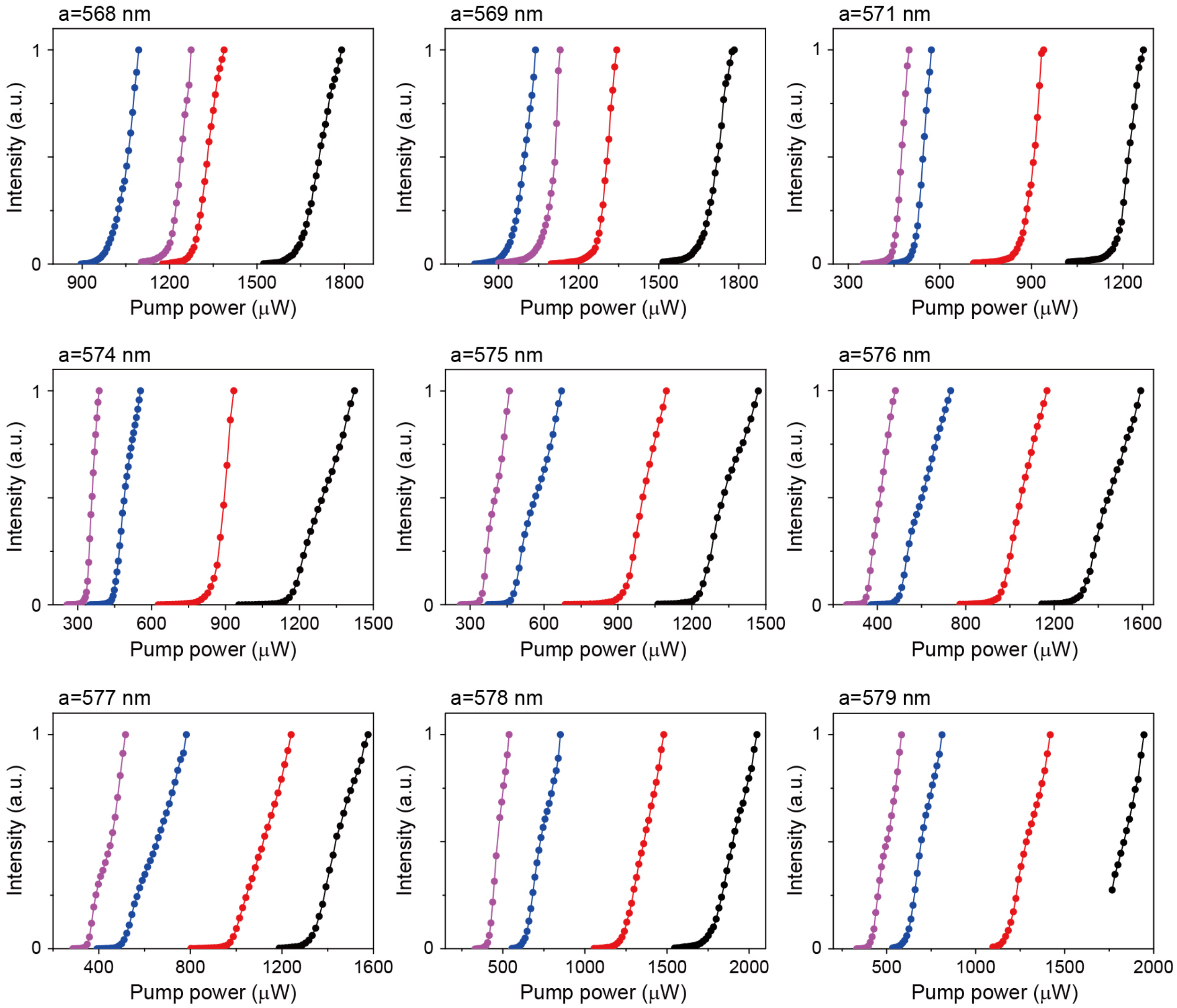

Supplementary Fig. 11. Measured L–L curves in laser structures with different lattice constants, with varying pump spot sizes. We examine four pump spot sizes: ~5.4 μm (purple), ~6.7 μm (blue), ~8.0 μm (red), and ~9.2 μm (black). The data in Fig. 4 were obtained with the pump spot size of ~5.4 μm. We note that mode competition is critical before the pre-merging (a = 568 and 569 nm).

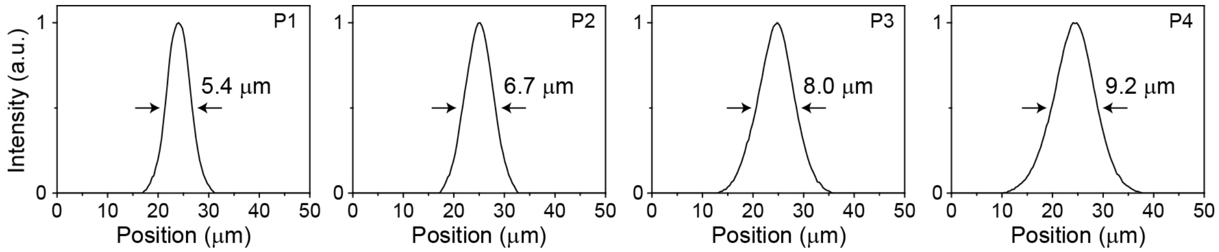

Supplementary Fig. 12. The estimate of the pump spot sizes. Four different pump spot sizes (P1 to P4) were used in the experiment (from left to right): ~5.4 μm, ~6.7 μm, ~8.0 μm, and ~9.2 μm.

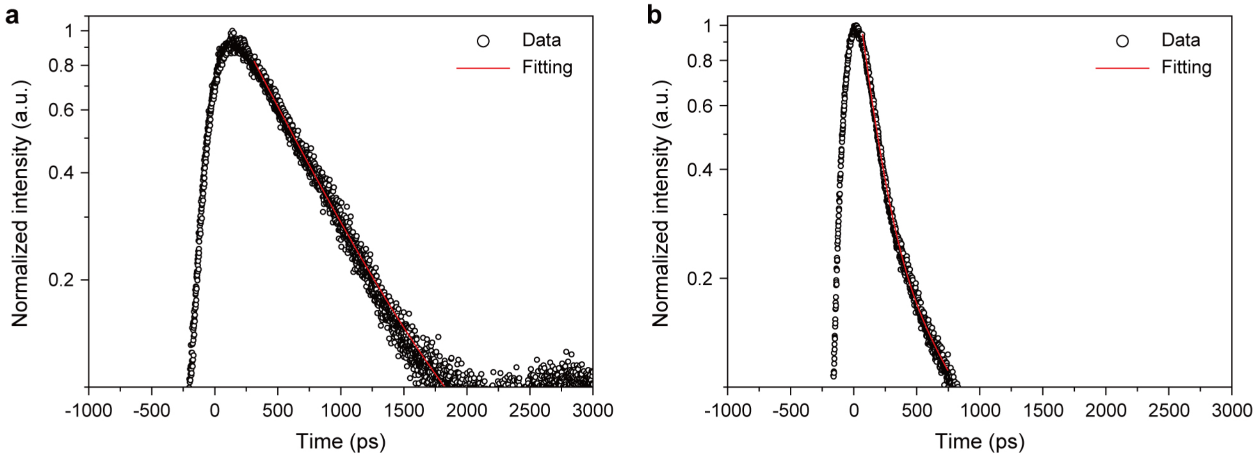

Supplementary Fig. 13. Time-resolved PL measurements in the spontaneous emission (**a**) and lasing (**b**) regions of the super-BIC laser. The solid red lines of spontaneous emission and lasing are fittings with decay times of 614 ± 4 ps and 138 ± 7 ps, respectively. We note that the decay time in lasing is limited by the measurement setup.

	Laser type	Threshold peak power (mW)	Threshold power density (kW/cm^2)	Q factor
This work	Super-BIC	0.34	1.47	>7300
Science 367, 1018 (2020)	BIC	5.28×10^5	4.2×10^4	-
Nano Lett. 20, 6005 (2020)	BIC	5.09×10^6	1.8×10^5	2590
Nature Nanotech. 13, 1042 (2018)	BIC	8.80×10^5	7.0×10^4	2750
Nature 541, 196 (2017)	BIC	15.6	~4	-
arXiv:1707.00181	BIC	~73	-	-
Nature Nanotech. 15, 67 (2020)	Topological	-	4.5	-
Science 359, eaar4005 (2018) *	Topological	~11000	~16	-

* The article does not explicitly mention whether it is peak power density or average power density.

Supplementary Table 1. Comparison of the super-BIC laser with other BIC and topological lasers.

Supplementary Note 1. Analysis of finite-size effects on the Q factor of BICs.

In this note, we analyze the finite-size effects on the Q factor of the fundamental TE-like mode in the vicinity of the Γ point. We are looking for the optimal position of accidental BIC, k_t, at which the Q factor of the finite-size structure is maximized. We start from the analytical dependence of Q factor on k-vector for an infinite-size photonic structure, which is isotropic in (k_x, k_y) space from general symmetry considerations,

$$Q(k)^{-1} = Q_0^{-1} k^2 (k - k_t)^2 (k + k_t)^2.$$

Here, Q_0^{-1} is the constant that is independent on k, and k_t is the distance from the origin to the accidental BIC. The equation above can be re-written as

$$Q(k_0)^{-1} = Q_0^{-1} \iint d^2k \delta(k_x - k_0) \delta(k_y - k_0) k^2 (k - k_t)^2 (k + k_t)^2.$$

Here we introduce delta-function to show that only the state with k = k_0 contributes to the Q factor, because there is no mixing of states within the band.

Next, we analyze the mode properties of finite-size but very large photonic structure with square lattice with the period a and number of unit cells $N_x = N_y = N >> 1$. The Q factor, Q_f, of modes of a finite-size structure can be evaluated by assuming that they radiate into channels i corresponding to the modes of the infinite-size structure averaged with certain weights α_i,

$$Q_f(k_0)^{-1} = \sum_{k_i} \alpha_i(k_i, k_0) Q(k_i)^{-1}.$$

Going from summation to integration, we get

$$Q_f(k_0)^{-1} = Q_0^{-1} \iint d^2k \alpha(k_x, k_y, k_0) k^2 (k - k_t)^2 (k + k_t)^2.$$

The weight function can be estimated as the delta sequence which tends to the delta function for infinite-size structures

$$\alpha(k-k_0)=\frac{1}{\pi\delta k^2}e^{-\frac{(k_x-k_0)^2}{\delta k^2}}e^{-\frac{(k_y-k_0)^2}{\delta k^2}},$$

where $\delta k_{\mathrm{x}} = \delta k_{\mathrm{y}} = \delta k$ is the parameter describing broadening of the delta-function and $\frac{1}{\delta k_{x,y}a}\sim N$>>1. We assume that the accidental BIC is located near the Γ point, so the integration in the right-hand side of the equation for $Q_f(k_0)^{-1}$ can be extended to the infinite domain because of a rapid decline of the exponential function.

We further integrate the right-hand side in the equation for $Q_f(k_0)^{-1}$ at the Γ point assuming $k_0 = 0$,

$$Q_f^{-1} = Q_f(0)^{-1} = Q_0^{-1}\delta k^2(6\delta k^4 - 4\delta k^2 k_t^2 + k_t^4).$$

This equation shows that the value of Q_f is maximized at the pre-merging regime for $k_t = \sqrt{2}\delta k$. The Q factor of the mode in the pre-merging regime at $k_t = \sqrt{2}\delta k$ is three times higher than for the merging condition $k_t = 0$.
